# Polyphenolic Profile and Targeted Bioactivity of Methanolic Extracts from Mediterranean Ethnomedicinal Plants on Human Cancer Cell Lines

**DOI:** 10.3390/molecules21040395

**Published:** 2016-03-23

**Authors:** Antonino Pollio, Armando Zarrelli, Valeria Romanucci, Alfredo Di Mauro, Federica Barra, Gabriele Pinto, Elvira Crescenzi, Emanuela Roscetto, Giuseppe Palumbo

**Affiliations:** 1 Department of Biology, University of Naples “Federico II”, Complesso di MS Angelo, 80126 Naples, Italy; 2 Department of Chemical Sciences, University of Naples “Federico II”, Complesso di MS Angelo, 80126 Naples, Italy; 3 Consorzio Interuniversitario Sannio Tech, P.zza San G. Moscati 8, SS Appia km 256, 82030 Apollosa (BN), Italy; 4 Department of Molecular Medicine and Medical Biotechnology, University of Naples “Federico II”, 80131 Naples, Italy; 5 Institute of Experimental Endocrinology and Oncology (IEOS), National Research Council (CNR), Via S. Pansini 5, 80131 Naples, Italy

**Keywords:** *J. communis*, *C. coggygria*, polyphenols, plant alcoholic extracts, cancer cells, cell cycle

## Abstract

The methanol extracts of the aerial part of four ethnomedicinal plants of Mediterranean region, two non-seed vascular plants, *Equisetum hyemale* L. and *Phyllitis scolopendrium* (L.) Newman, and two Spermatophyta, *Juniperus communis* L. (*J. communis*) and *Cotinus coggygria* Scop. (*C. coggygria*), were screened against four human cells lines (A549, MCF7, TK6 and U937). Only the extracts of *J. communis* and *C. coggygria* showed marked cytotoxic effects, affecting both cell morphology and growth. A dose-dependent effect of these two extracts was also observed on the cell cycle distribution. Incubation of all the cell lines in a medium containing *J. communis* extract determined a remarkable accumulation of cells in the G2/M phase, whereas the *C. coggygria* extract induced a significant increase in the percentage of G1 cells. The novelty of our findings stands on the observation that the two extracts, consistently, elicited coherent effects on the cell cycle in four cell lines, independently from their phenotype, as two of them have epithelial origin and grow adherent and two are lymphoblastoid and grow in suspension. Even the expression profiles of several proteins regulating cell cycle progression and cell death were affected by both extracts. LC-MS investigation of methanol extract of *C*. *coggygria* led to the identification of twelve flavonoids (compounds **1**–**11**, **19**) and eight polyphenols derivatives (**12**–**18**, **20**), while in *J*. *communis* extract, eight flavonoids (**21**–**28**), a α-ionone glycoside (**29**) and a lignin (**30**) were found. Although many of these compounds have interesting individual biological activities, their natural blends seem to exert specific effects on the proliferation of cell lines either growing adherent or in suspension, suggesting potential use in fighting cancer.

## 1. Introduction

Many natural products have biological activity that can be of therapeutic benefit in treating diseases [1]. Indeed, during the very last years, after the advent of molecular biology and combinatorial chemistry, natural products have partially lost their edges, assuming a secondary role in drug discovery and drug development. However, more recent times have seen a rejuvenated and renewed interest for natural compounds and their role as powerful basis for drug development. The reason to search for pharmaceutically active plant extracts is very likely related to the co-evolution of plants and animals: it is not surprising that plants have developed specific strategies aimed to defend themselves from predation also producing substances able to interfere (negatively) with the animals physiology. Plant extracts can act as potent muscle relaxants emetics, affect heart physiology, possess antimicrobial properties, interfere with or hamper the cell cycle, and some are endowed of anticancer properties [2], often displaying a synergistic action that increases their effects [3]. In this regard, in particular, the knowledge of the cytological mechanisms involved in their actions seems of the utmost importance for an eventual precise evaluation and development. This study originated from previous observations made in this laboratory, searching for antimicrobial properties of plant extracts [4]. In that case, we considered only extracts that resulted active against selected bacteria but were void of cytotoxicity. Now, on the contrary, we have focused our attention on four extracts, two from non-seed vascular plants, *Equisetum hyemale* L. (*E. hyemale*) and *Phyllitis scolopendrium* (L.) Newman (*P. scolopendrium*), and two from Spermatophyta, *Juniperus communis* L. (*J. communis*) and *Cotinus coggygria* Scop (*C. coggygria*), that, in turn, were characterized by some cytotoxicity. In this regard, we present new data on the biological effect of these extracts. We demonstrate that the alcoholic extracts of *C. coggygria* and *J. communis* possess interesting and reproducible properties that may merit further attention as they were able to alter, each one with a specific effect, the cell cycle of four human cancer cell lines, independently from the cells phenotype and origin. Two of them have epithelial origin (A549 and MCF-7, from lung and breast adenocarcinomas) and two are lymphoblastoid (U936 and TK6). The two epithelial cells grow adherent to the plate surfaces, while the two lymphoblastoid cells grow in suspension. Several chemical analyses of the extracts from the active plants were performed allowing the isolation and identification of several flavonoids and polyphenol derivatives.

## 2. Results and Discussion

We have studied the effects of several plant extracts on four human cell lines, namely MCF-7 (breast cancer), A549 (lung adenocarcinoma), U-937 (histiocytic lymphoma) and TK6 (human B lymphoblastoid cells). The first two cell lines are anchorage-dependent, while the second two grow in suspension. In order to evaluate the *in vitro* cytotoxic potential of the plant extracts, the effects of different dilutions of each extracts were first analyzed by Trypan Blue exclusion assay, on the adherent cell lines (data not shown). Analysis of these data allowed for the selection of the appropriate dilution of the extracts. In addition, evident cellular effects (partial detachment, floating and changes in morphology) were observed incubating MCF7 and A549 cells with extract from *J. communis* or *C. coggygria*. These effects were much less pronounced, when cells were incubated with diluted extracts from *E. hyemale* or *P. scolopendrium*. Hence, while focusing our attention on *J. communis* or *C. coggygria*, we have used, when needed, the extracts from *E. hyemale* or *P. scolopendrium,* as controls.

### 2.1. Cell Viability and Growth

**Figure 1:**
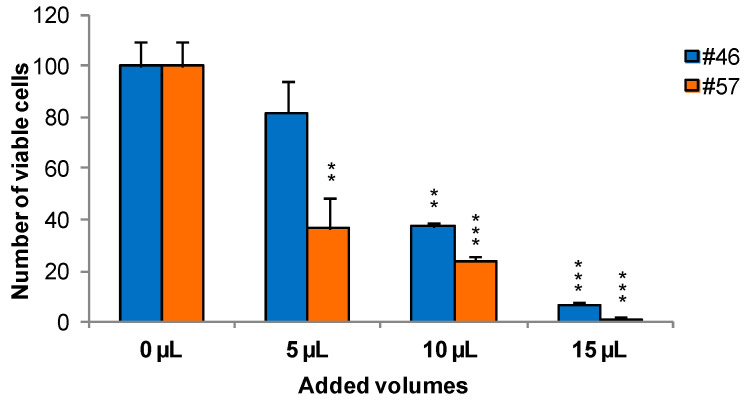
Twenty-four hours of incubation with increasing amounts of extracts #46 and #57 arrest the growth of MCF-7 cells in a concentration dependent manner (*p* values: ** < 0.01; *** < 0.001).

To measure the effects of extracts on cells viability and growth, MCF-7 was selected as an illustrative example cell line. Cells were treated for 24 h with extracts #46 (*J. communis*) and #57 (*C. coggygria*), at 0.05%, 0.1%, and 0.15% *v*/*v*; the concentrations are expressed as percent *v*/*v* of the saturated solutions at room temperature (*i.e*., a solution that contains the maximum concentration of solutes. Additional solute will not dissolve in a saturated solution) as also reiterated in Section 3.2. At the end of this incubation, Trypan blue solution was added and the cells were counted using a hemocytometer. Data indicate that extracts affect cell viability in a dose-dependent manner. In addition, it appears that extract #57 is more toxic to cells than extract #46 (Figure 1).

**Figure 2:**
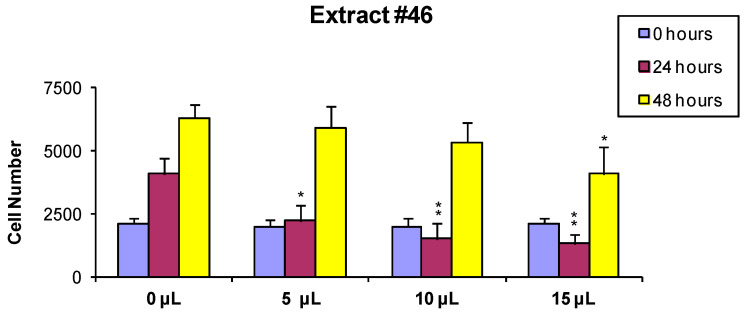
MCF-7 cells were treated for 24 h with 0 (controls), 5, 10 and 15 µL/mL of the extract #46, washed and counted. Two thousand cells from each incubation condition were seeded in a fresh medium-extract-free and counted again 24 and 48 h later (*p* values: * < 0.1; ** < 0.01).

**Figure 3:**
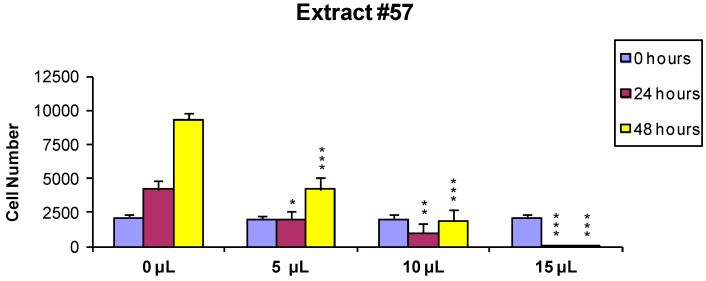
MCF-7 cells were treated for 24 h with 0 (controls), 5, 10 and 15 µL/mL of the extract #57, washed and counted. Two thousand cells from each incubation condition were seeded in a fresh medium-extract-free and counted again 24 and 48 h later (*p* values: * < 0.1; ** < 0.01; *** < 0.001).

In order to evaluate if the observed cytotoxic effects were reversible or irreversible, MCF7 cells were incubated for 24 h with the extracts, as described above, and the cells surviving the treatment were washed and released in fresh medium, and further analyzed 24 and 48 h later As shown in Figure 2 and Figure 3, the effect of extract #46 on cell proliferation was reversible, while that of extract #57 was irreversible at higher concentration.

These data, on the whole, demonstrate that both *J. communis* and *C. coggygria* extracts affect cell viability in a dose- and time-dependent manner. The extract #57, in addition, when used at elevated concentration (≥15 µL/mL) induces an irreversible growth arrest and cell death.

### 2.2. Effects of the Extracts on Cell Cycle

We first studied the alterations in the cell cycle profile of A549 cells treated with the plant extracts for 24 h. We show data obtained with the highest life-compatible concentration explored (0.15% *v*/*v*). In such conditions, the cell cycle profiles displayed significant alterations.

**Figure 4:**
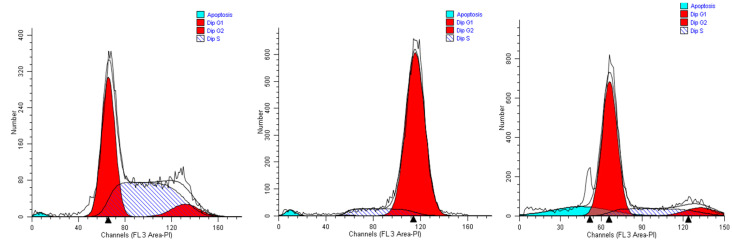
Representative cell cycle profiles of untreated A549 cells (controls, **left**), and cells incubated in media containing 0.15% (*v*/*v*) of extracts #46 (**middle**) and #57 (**right**).

When A549 cells were incubated with the extract #46 for 24 h, a significant number of cells detached, while the others changed morphology assuming a rounded shape. Accordingly, cell cycle analyses showed an accumulation of cells in the G2/M phase (from <8% in normal proliferating cells to about 90% in extract #46-treated cells) (Figure 4, middle panel) This effect was reversible as, replacing the cells media with fresh media (not containing the extracts), cells regain a normal phenotype (morphology) and then appear to regain their grow potential.

In contrast, when the same cells were treated with extract #57 at low concentrations (*i.e.*, 0.15% (*v*/*v*), an accumulation of cells in G1 phase of cell cycle was observed (+20% as compared to controls), associated with a reduction of cells in S phase (Figure 4, right panel). Even in this case, replacing the media, the cells return cycling normally.

**Figure 5:**
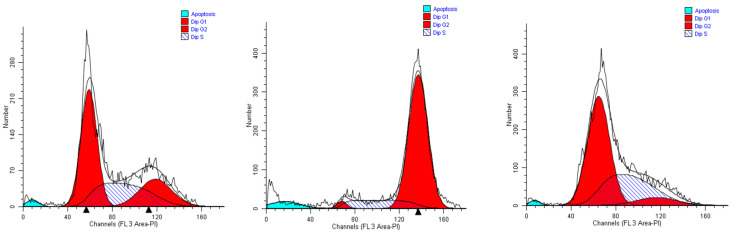
Representative cell cycle profiles of untreated MCF-7 cells (controls, **left**), and cells incubated in media containing 0.15% (*v*/*v*) of extracts #46 (**middle**) and #57 (**right**).

As the MCF-7 cells are concerned, we obtained results quite completely superimposable to those gained with A549 cells when incubated with extract #46 (0.15%, *v*/*v*). Most of the cells accrual in the G2M phase, reaching 82% of the total. Considering that in control cells (Figure 5, left panel) the population in this phase does not exceed 30%, extract #46 induces an increase superior to 50% (Figure 5, middle panel). The addition of extract #57 to cell medium for 24 h induced a substantial increase in the percentage of resting cells (G0/G1), as the increase amounts to near 25% (Figure 5, right panel).

Overall, the cytometric data presented above refer principally to two different epithelial cancer lines that, although transformed are p53 positive and grow adhering to the surface of culture plates. Both sets of data, indeed, suggest that the extracts #46 and #57, individually, cause in the two cell lines similar and coherent effects. In particular, it appears that extract #46 arrests the cycle in the G2/M phase, while the extract #57 blocks the cells in the G1 phase.

These observations prompted us to verify if the specific effects produced by the two extracts on adherent cells could be reproduced also on cells growing in suspension. Indeed, most of the data obtained in this regard are concerned with the U937 cell line, but these findings are also supported by some experiments on TK-6 cells (not shown).

**Figure 6:**
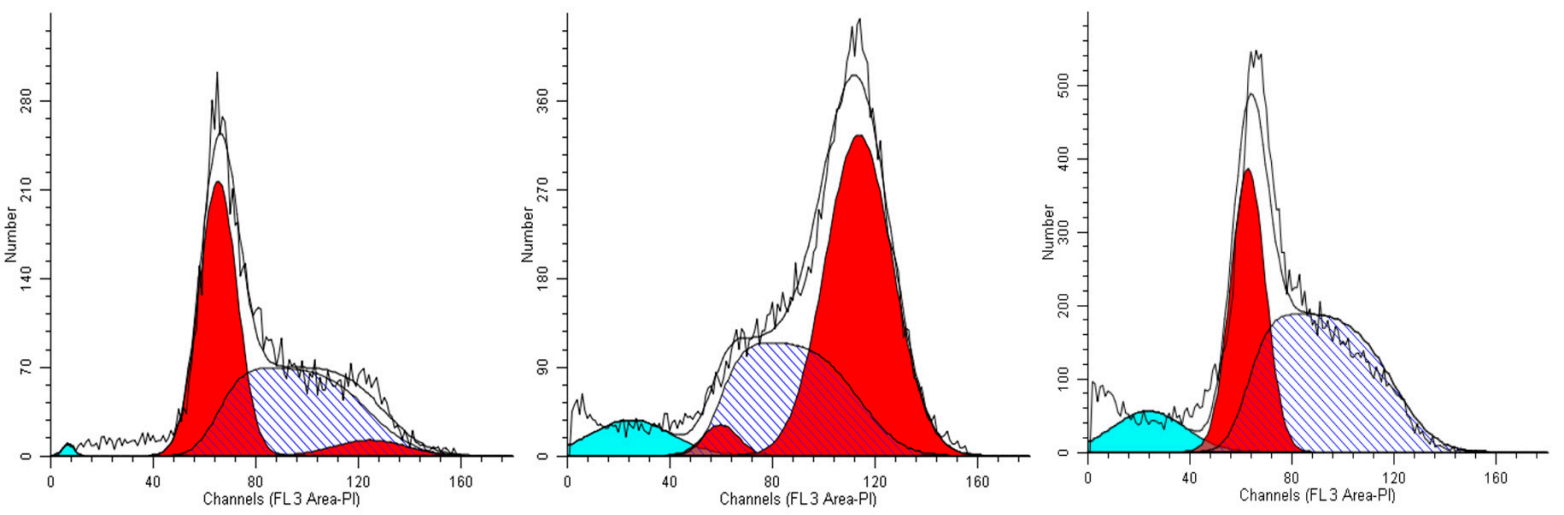
The cell cycle profiles of untreated U-937 cells (controls, **left**), and cells incubated for 24 h in media containing 0.15% (*v*/*v*) of extracts #46 (**middle**) and #57 (**right**).

Twenty-four hours of incubation of U937 cells in a medium containing 0.15% (*v*/*v*) of extract #46 determined a remarkable accumulation of cells in the G2/M phase. The percentage of cells populating this phase in the controls amounted to about 5% of the total (Figure 6, left panel), while after the incubation the same population was increased to more than 60% (Figure 6, middle panel).

The addition of extract #57 (0.15%, *v*/*v*) to cell medium for 24 h induced a visible increase in the percentage of G0/G1 cells (compare the left and right panels of Figure 6). The addition of both extracts #46 and #57, however, also causes the formation of debris (non-specific cell fragments and possibly apoptosis).

Also TK-6 cells were incubated with extracts #46 and #57 producing the same type of effect as observed in the other cell strains (not shown).

The treatment of the various cell lines for 24 h with other extracts, namely #29 (*E. hyemale*) and #32 (*P. scolopendrium*), did not affect the cell cycle profiles (Figure S1–3) as well as their morphology and viability. This demonstrates that the active principles interfering with the cell cycle of various cells are specific of the particular plant extract.

Until now, only limited data about molecular events underlying the cell cycle perturbation caused by exposure of cells to *C. coggygria* and *J. communis* extracts were available. Most of the findings reported are concerned with the effects on cell counting and residual cell viability [5,6].

### 2.3. Protein Expression

Even less information on the changes in the expression of specific proteins by cells treated with the extracts from the two plants has been reported.

Changes in the proliferation rate of cells is a controlled process in which some proteins play pivotal roles arresting or allowing cell cycle progression between successive phases. It is known, in fact, that the inhibition of cell proliferation, in general caused by the extracts observed should be echoed in the expression of some proteins involved in cell cycle progression and/or arrest and cell final fate. These proteins may also mediate the manifestation of premature cellular senescence. This process is an ensemble of complex biological processes some of which have apparently opposing effects (repair, tumor suppression/promotion, and aging).

For this reason, we were aimed to study the changes in the expression levels of a panel of these proteins when cells were exposed to non-lethal doses of the extracts. Only a limited number of such proteins, namely Cyclin B, Cdk1, Cdk2, p53, p21, p27, Caspase 9, Bcl-XL and Bcl-2, were actually analyzed and principally (but not exclusively) in MCF-7 and A549 cells.

#### 2.3.1. Cyclin B, Cdk 1

**Figure 7:**
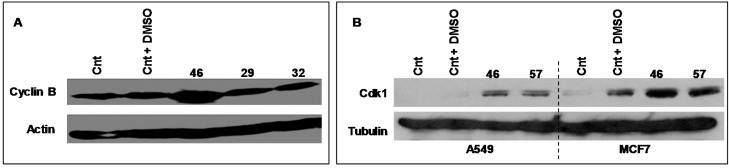
The pictures illustrate the electrophoretic expression profiles of Cyclin B (**A**, MCF7 only) and Cdk1 (**B**) of A549 and MCF-7 cells exposed for 24 h to extracts #46, #29 and #32 (0.15%, *v*/*v*). Both panels indicate that this exposure increases the expression of Cyclic B and Cdk-1 proteins. Tubulin (**B**) and actin (**A**) have been used as loading controls, respectively.

Cyclic B and Cdk1 are proteins involved in the cell cycle, generally acknowledged as pivotal regulators of cell cycle during the transition from the G2 to M phase. Figure 7 illustrates the electrophoretic expression profiles of Cyclin B and Cdk1 of MCF-7 cells exposed for 24 h to extract #46 (0.15%, *v*/*v*). The clear augment of expression of both Cyclin B and CDK1 is in accord with the experimentally observed arrest of cell cycle.

In the Figure 7A is also shown the expression profile of Cyclin B of MCF-7 cells exposed to extracts #29 (*E. hyemale*) and #32 (*P. scolopendrium*) that do not alter the cell cycle of all cells studied and are used as negative controls.

#### 2.3.2. p53

**Figure 8:**
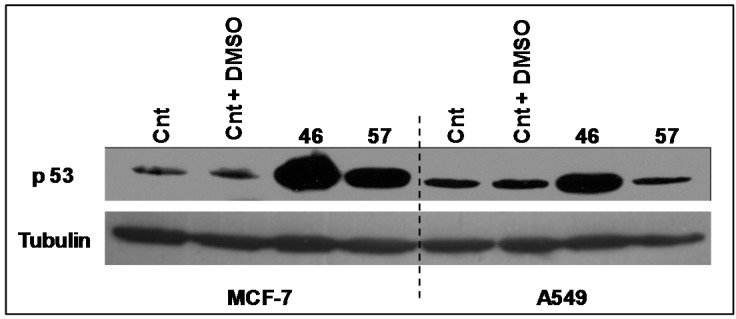
Changes in the expression of p53 in A549 and MCF-7 cells induced by extracts #57 and #46. Extract #57 increases the expression of p53 exclusively in MCF-7 cells. Tubulin has been used as loading control.

The expression levels of the transcriptional factor p53 (Figure 8) have been analyzed in A549 and MCF-7 incubated with extracts #46 and #57. It appears that the arrest in the G2/M phase induced by extract #46 is accompanied by an increase in p53 expression in both A549 and MCF-7 cells. Differently, extract #57 induces the expression of p53 exclusively in MCF-7 cells.

#### 2.3.3. Cdk2, p21 and p27

The protein Cdk2 is indispensable for the G1/S transition exerting its action through its association with specific regulatory cyclins. When required by specific conditions, Cdk2 function may be reduced or abolished by interacting with the protein p21, a specific inhibitor [7]. p27 might also play different roles in the cell cycle. Its over expression, for instance, regulates growth and differentiation, while moderate expression induces cell cycle arrest [7,8,9,10].

**Figure 9:**
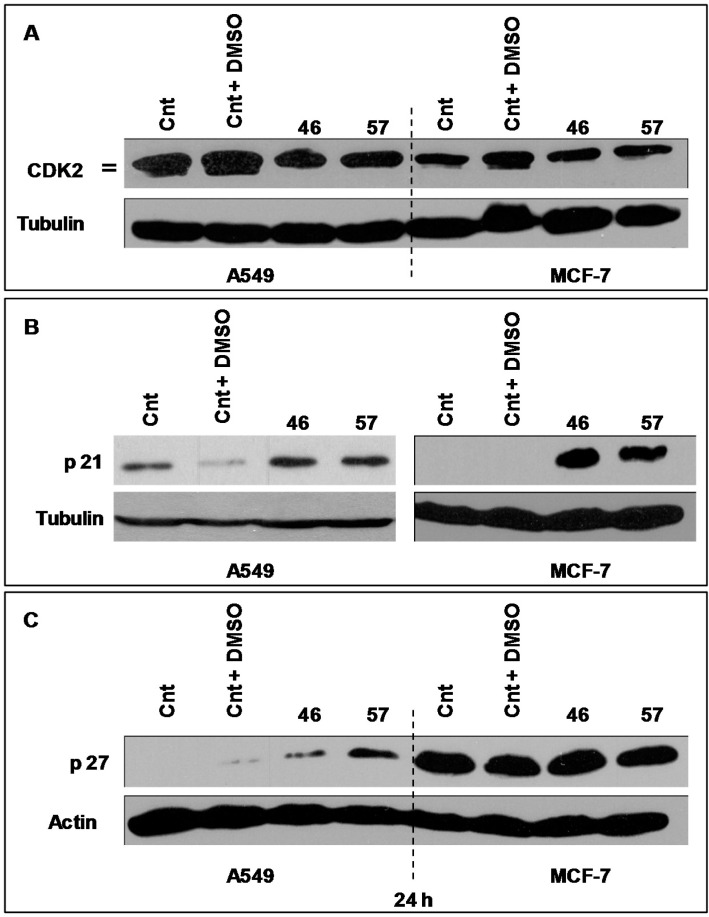
(**A**) Changes in the expression of CDK2 in A549 and MCF-7 cells induced by extracts #57 and #46. The CDK2 doublet visible in the controls fades with the addition of both extracts indicating a decline in the kinase activity. Tubulin has been used as loading control; (**B**) p21 expression in MCF-7 is induced by extracts #46 and #57. Actin has been used as loading control; (**C**) p27 expression (after 24 h) in A549 and MCF-7 cells treated with extracts #46 and #57. p27 protein is significantly induced by both extracts in A549 cells, but not in MCF-7 cells. Actin has been used as loading control. p21 ((**B**), right panel) and p27 ((**C**), right panel) expression in MCF-7 cells were probed from the same blot, and the same actin blot is shown as a control for both p21 and p27.

The expression of Cdk2 by A549 (Figure 9A, left) and MCF-7 cells (Figure 9, right) treated with extracts #46 and #57 changes as the electrophoretic doublet, present in controls, tends to fade following a decrease in the kinase activity.

As p21 is concerned, we have analyzed its expression in both MCF-7 and A549 cells (Figure 9B). In this case, it appears that, compared to controls, p21 is strongly expressed in MCF-7 cells, while no evident changes have been observed in A549 cells.

Similarly we have analyzed the expression profiles of p27, a protein that may also block the cycle progression at the level of the G1/S transition. Figure 9C depicts the expression of p27 in A549 and MCF7 treated with extracts #46 and #57. It appears that A549 cells are more sensitive to extracts than MCF-7 as A549 cells do not express appreciable basal levels of p27. On the contrary, the addition of either extracts #46 or #57 (the latter in particular) results in substantial increase of expression levels of this protein. This effect was not observed in MCF-7 cells, whose p27 basal level is already sustained. Then, the addition of extract #57 does not cause any appreciable augment of p27 expression.

#### 2.3.4. Bcl-XL

**Figure 10:**
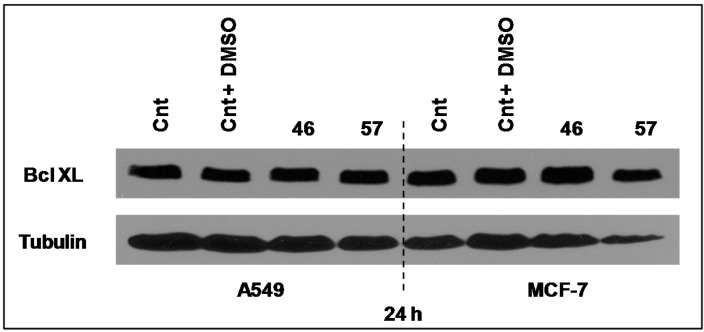
Extracts #46 and #57 do not appear to alter the expression levels of Bcl-XL in MCF-7 and A549 cells.

Bcl-XL is a member of Bcl2 family proteins that is located within the mitochondrial membrane. Its pro-survival action is founded on its capacity to prevent Cytochrome C release and the associated caspases activation [11]. However, its expression level in both MCF-7 and A549 cells after 24 h of incubation with extracts #46 and #57 did not appear to sensibly change (Figure 10).

We did not investigate in detail the potential anti-/pro-apoptotic effects exerted by the extracts on cell lines. Our observations are limited to changes in Bcl-2 expression and Caspase 9 activation in the MCF-7 cells only by extract #46. These data, while indicating the absence of anti-apoptotic effects (Figure S4), suggest that the expression of Caspase 9 was scarcely altered within 24 h. Indeed, only at the highest extract concentration (*i.e.*, 15 μg/mL), the expression of Caspase 9 appears to be visibly reduced indicating a possible initiation of an apoptotic process. This observation is reported in Figure S5.

### 2.4. Evaluation of Senescence in Cells

**Figure 11:**
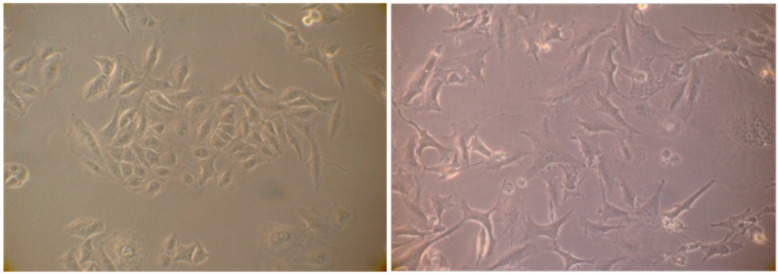
(**left**) Normal proliferating A549 cells; and (**right**) senescent A549 cells 72 h after treatment with the extract. Note the volume increase of cells, the cytoplasm flattening and the presence of numerous vacuoles.

**Figure 12:**
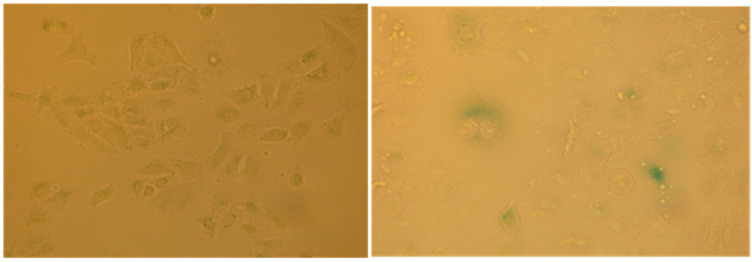
β-Galactosidase activity in senescent A549 cells: (**left**) negative control at pH 6; and (**right**) cells treated with extract #57 and then analyzed 72 h later, as described in the text.

The cell cycle arrest in G1 phase, determined by the action of some agents including chemotherapy may induce in cells premature senescence [12] an ensemble of complex biological processes characterized by cells specific morphological and biochemical changes. The effects produced by the extract #57, which compels most cells into G1 phase, prompted us to verify if it would induce such condition in A549 cells that are prone to become senescent in stressing condition. To this purpose, A549 cells were incubated for six days in the presence of 5 µL/mL of extract #57 of medium (every two days, the medium was replaced with fresh medium containing the same amount of extract). Cells were then incubated for additional 72 h with fresh medium without the extract. At the end of this treatment, cells were analyzed for morphological changes (Figure 11) and for the acquired capacity to express SA-β-galactosidase activity (Figure 12), through the appearance of the characteristic green-blue spots in the cytoplasm.

This finding indicates that the extract #57 at a non-lethal concentration promotes specific G1 accumulation and induce premature senescence, a characteristic effects associated to anticancer drugs when used at low concentrations (doxorubicin, for example).

### 2.5. DPPH-Based Assay to Estimate the Antioxidant Capacity of J. commnunis and C. coggygria Extracts

The alcoholic extracts of the aerial parts of *J. communis* and *C. coggygria* plants were tested for their scavenging effects towards 2,2-diphenyl-1-picrylhydrazyl (DPPH) radical. We have found that *J. communis* extract was endowed of a good antiradical activity, as it is able to scavenge the DPPH• by 57.5% ± 2.3% at a dose of 100.0 μg/mL. At the same concentration, *C. coggygria* extract was much less powerful as its scavenging capacity was about 50% of that of extract from *J. communis*.

### 2.6. Identification and Characterization of Chemical Components of the Plant Extracts

**Figure A1:**
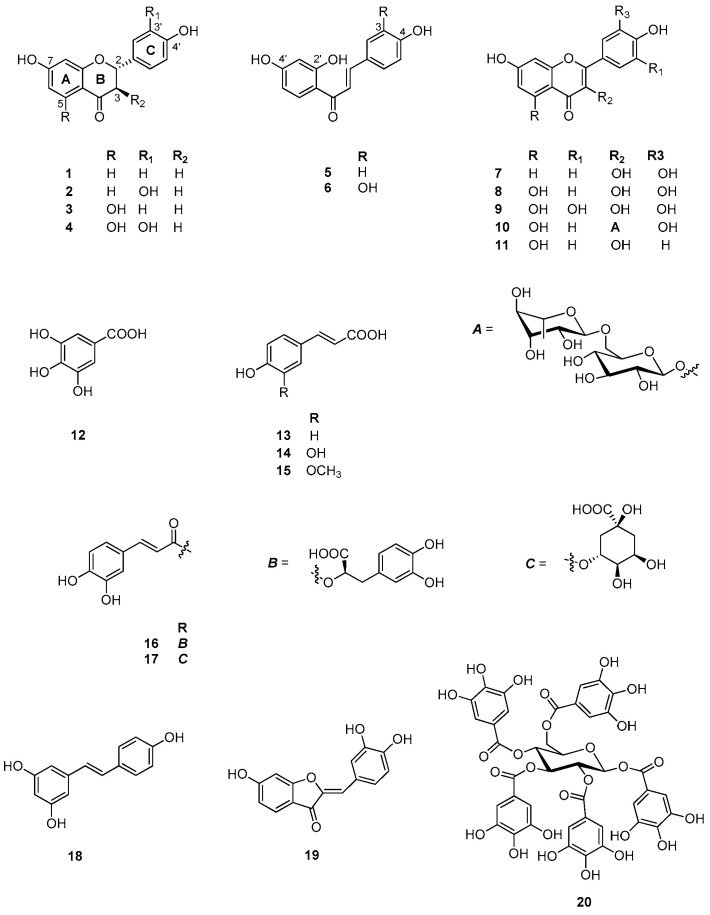
Chemical structures of compounds **1**–**20** isolated from *C. coggygria*.

**Figure A2:**
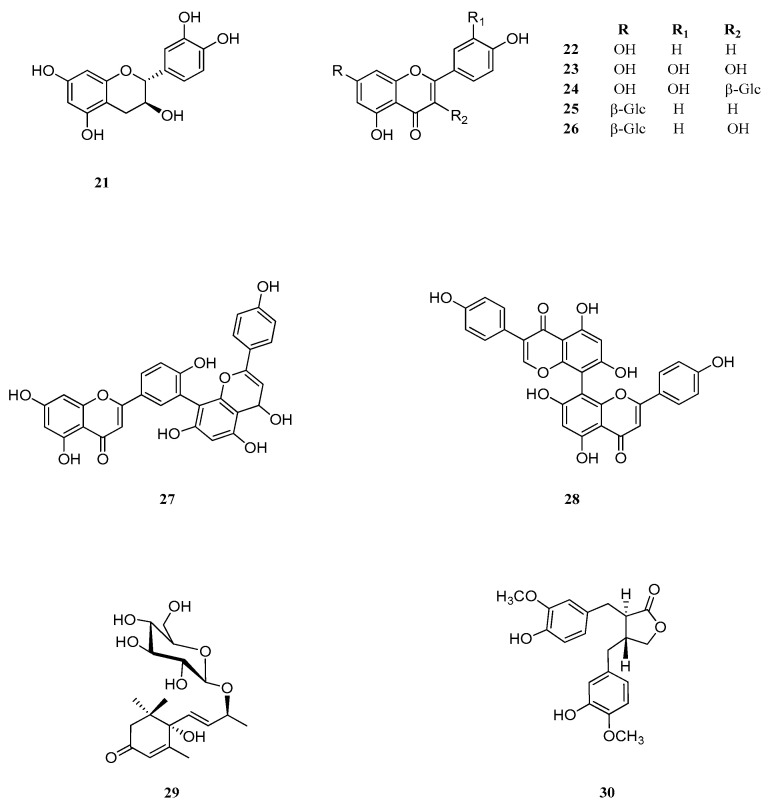
Chemical structures of compounds **21**–**30** isolated from *J. communis*.

The last aspect that we have considered was the identification of the major natural components of the plant extracts. The Table 1 summarizes the array of phenolic compounds that we have identified in methanolic extracts of *C. coggygria* and *J. communis* by liquid chromatography–mass spectrometry (LC-MS) detection. In particular, LC-MS investigation of methanol extract of *C. coggygria* led to the identification of flavonoids **1**–**11** and **19** and polyphenols derivatives **12**–**18** and **20**, while in *J. communis* methanol extract the flavonoids **21**–**28**, the α-ionone glycoside **29** and the lignin **30** were found. All isolated compounds were identified for comparison with the data reported in the literature and in the database of the instrument. Their chemical structures are shown in Figure A1 and Figure A2 whereas the IUPAC names are indicated in Table A1 and Table A2 of the Appendix A.

Most of them certainly hold biological properties that may interfere with cell proliferation as suggested by Kuntz *et al*. [13]. For instance, as far as flavonoids are concerned, fisetin, sulfuretin, isoliquiritigenin, quercetin, apigetrin, and amentoflavone [14,15,16,17,18,19,20] have all been considered active against breast cancer cells acting at various levels.

Similarly, polyphenol derivatives isolated from *C. coggygria* and *J. communis* have shown cytotoxic activity against several cancer cell lines: caffeic, coumaric and ferulic acids can act against lung and colon cancer cell line growth, controlling adhesion and migration steps of tumor progression [21], and penta-*O*-galloylglucose [22], inhibit the proliferation of human breast cancer cells. The anti-proliferative action is not limited to simple phenolics: also the lignan matairesinol evidenced a cytostatic activity against breast and colon cancers [23].

Polyphenols derivatives may cause cell cycle arrest and induce apoptosis; resveratrol was shown to activate p53 by phosphorylation, and significantly increases the pro-apoptotic signals such as Bak and p21 genes [24,25], whereas Gallic acid stimulated the synthesis of Bax and Bad protein and induced a decrease of Bcl-2 and Bcl-xL levels in NCI-H460 lung cancer cells [26]. But also flavonoids as quercetin affect the cell cycle of different cancer cell lines, inhibiting the activity of CDK2 [27], activating caspases and downregulating the expression of anti-apoptotic Bcl-2 and tumor suppressing p53 proteins [28]. In the same way, apigenin and kampferol can induce a strong down-regulation of CDK1 and a relatively small up-regulation of p27 [29]. Both extracts that we studied possess mild to weak antioxidant properties. This may result by the concomitant presence of several natural substances whose individual characteristics may differ from those of the blend that we find in the extract. Natural products, and particularly those possessing considerable variation of phenolic composition certainly have a steric complexity larger than any synthetic drugs [30]. This implies that plant crude extracts may present unpredictable therapeutic effects that are different, in type and intensity, from those exhibited by individual components [31].

Undoubtedly, the extracts own attractive and unique properties that may find interesting applications for *in vitro* studies and even as potential drugs. This, however, deserves further attention and requires more targeted studies.

## 3. Materials and Methods

### 3.1. Collection of Plant Samples

*C. coggygria*, *E. hyemale*, *J. communis* and *P. scolopendrium* plants were collected during spring and summer of 2014 in the regional parks of Matese and Cilento, Campania, Southern Italy, by scientists from the Dipartimento delle Scienze Biologiche of the University Federico II of Naples. Shortly after collection, plant materials (branches and leaves) were air-dried, cut to slices and finely crushed. The resulting powder-like materials were stored at −20 °C. Voucher specimens have been deposited in the Herbarium of the Botanical Garden of the “Federico II” University in Naples, Italy.

### 3.2. Preparation of Plant Extract

Exactly 4 g of each powdered plant materials (*i.e.* from the above 4 plants) were all extracted with the same procedure as previously described [4]. In brief, extraction procedure was carried out at room temperature (~25 °C) in 100 mL Erlenmeyer flasks. Suspensions were kept in ultrasonic baths for 30 min, followed by 24 h continuous stirring (90 rpm) in a rotary shaker. Extracts were then filtered on paper (Whatman, n.1) and concentrated using a vacuum roto-evaporator at 38 °C. The dried material was finally weighted and dispersed in 1 mL of 10% aqueous dimethylsulfoxide (DMSO). The suspensions, kept at 37 °C overnight under gentle stirring, were finally centrifuged in a microfuge for 5 minutes. Centrifuged samples showed a substantial solid separated phase (insoluble residue) at the bottom overlaid with a transparent solution. These intensely colored (from deep green to orange/brown) saturated solutions were considered the “stock solutions” and stored at 4 °C in the dark. Before each use, stock solutions were brought to room temperature under continuous shaking to resuspend the solid material present at the bottom of the tubes. These suspensions were then transferred into Eppendorf tubes and microfuged extensively. Small amounts of liquids were collected from the surface of the clear solutions and finally diluted 1:10 with 2% DMSO. The final concentrations (expressed in percent *v*/*v*) of plant extracts in the medium of cells in the various experiments were of 0.005, 0.01 and 0.015 volumes of original “stock saturated solutions” for 100 volumes of cells culture media. In all experiments, the final DMSO concentration was always ≤1% *v*/*v*.

No special precautions were taken in handling stock solutions of extracts as their properties remained intact for longer than six months (in closed Eppendorf tubes, the dark at 4 °C). The extracts high concentrations, the high concentration of DMSO (which is characterized by intrinsic strong antioxidant properties), the small amount of air in contact with the solution surfaces within the small containers, and the rapid handling of samples all concurred to prevent or minimize oxidation.

### 3.3. General Composition of Plant Extracts

A detailed description of procedures used to isolate and identify the major natural components from plant extracts indicated below, is reported detailed in details in the Supplementary Materials Section of this work.

### 3.4. Microbiological Assay

Although extraction procedures were conducted with care and required a long stay of plants remnants in alcohol, sterility was not fully guaranteed. For this reason we performed a series of microbiological assays to test the presence of bacterial or mold contaminants in the various extracts. To this purpose we added 15 µL of each stock (very concentrated solutions) to plates (60 mm) containing 1 mL DMEM (control) or 1 mL DMEM containing 1 µL penicillin (100 µg/mL), 1 µL Streptomicin (100 µg/mL) and 1 µL Anfotericin B (2.5 µg/mL). Plates were incubated up to 72 h at 37 °C. Extracts from *J. communis* were found to be sterile, while a slight contamination was found in extracts from *E. hyemale*, *P. scolopendrium* (after 72 h of incubation) and *C. coggygria* (after 24 h of incubation). We then proceeded to identification of bacterial contaminants that were as indicated in Table 2.

The same amounts of extracts in the presence of antibiotics, as indicated above, showed full absence of bacterial growth.

### 3.5. Cell Lines

The cell lines used in this work are four, two having epithelial origin as the MCF-7 (human breast cancer) and the A549 (human lung adenocarcinoma and two lymphoblastoid lines, namely the U-937 (histiocytic lymphoma) and TK6 cells. The latter line, however, has been used only in limited sets of experiments. All cell lines have been obtained from ATCC (Rockville, MD, USA) and cultured according to its instructions. In brief, the two epithelial cell lines MCF7 and A549 cells have been cultured at 37 °C with 5% CO_2_ in 100 mm plates in Dulbecco’s Modified Eagle Medium (DMEM, Sigma, Milan, Italy) containing 20 mM Glycine. The U937 and TK6 cells, which grow in suspension, have been cultured in flasks in RPMI 1640–Glutamine. All media were supplemented with 10% FCS. At 70%–80% confluence, cells were detached by plates by trypsinization (MCF7 and A549) or centrifuged for 5 min at 1200 RPM (U937 and TK6) and re-plated or resuspended in fresh medium according to the needs. All cell media contained 1% penicillin/streptomycin/anfotericin.

### 3.6. Cells Growth and Viability Assay

Cells were plated in triplicate at 2 × 10^4^ per well in a 96-well plates. After 16 h, extracts were added to cells in the medium at 3 different concentrations, namely 0.05%, 0.1% and 0.15% (*v*/*v*). The final DMSO concentration in these cells was always set at <1%. Cell number was assessed using a hemocytometer. Trypan blue (0.08%; Sigma) was added to evaluate cell viability.

### 3.7. Flow Cytometry

Flow cytometric analysis was as described before [32]. In brief, about 1.5 × 10^5^ cells were harvested, fixed in 70% cold ethanol and stored at 4 °C overnight. Before analysis, cells were washed in distilled water, centrifuged and resuspended in 1 mL PBS containing propidium iodide (50 µg/mL, Sigma) and RNase-DNase-free (100 µg/mL, Roche, Milan, Italy), for DNA staining. After 1 h in the incubator at 37 °C, samples were kept in the dark for 20 min at room temperature before final readings. Fluorescence was detected using the 488 nm laser line with a CyAn ADP Flow Cytometer (DAKO Cytomation). About 20,000 events (≥15,000 cells) were recorded for each sample (in triplicate). Cell cycle profiles were analyzed using ModFit/LT 3.2 version (Verity Software, Topsham, ME, USA). Typical profiles (selected among two or three independent experiments) are reported in Figure 4, Figure 5 and Figure 6.

### 3.8. Western Blot Analysis

Total cell proteins preparations were obtained lysing an adequate number of cells by 1 mmol/L EDTA, 0.2% Triton X-100, 1 µg/mL aprotinin, 170 µg/mL phenylmethylsulfonyl fluoride, and phosphatase inhibitors (Sigma). Protein concentration was routinely measured by the Bio-Rad protein assay (Milan, Italy) [33]. Polyacrylamide gels (7.5%–15%) were prepared essentially as described by Laemmli [34]. Molecular weight standards were from New England Biolabs (Hitchin, Herts, UK). Proteins separated on the polyacrylamide gels were blotted onto nitrocellulose filters (Hybond-C, Amersham, Milan, Italy). Filters were washed and stained with specific primary antibodies and then with secondary antisera conjugated with horseradish peroxidase diluted 1:2000 (Bio-Rad). Filters were developed using the enhanced chemiluminescence Western blotting detection reagent (Amersham). Membranes were stripped and re-probed with different antibodies. Typical electrophoretic patterns (selected among two or three independent experiments) are reported in Figure 7, Figure 8, Figure 9 and Figure 10.

### 3.9. Antibodies

Anti-Bcl-2 (100), -p27KIP1 (C-19), -p21CIP1 (C-19), -Cdk2 (M2), -Cdk1, -p53 (DO-1), and -caspase-9 and all secondary antibodies were purchased from Santa Cruz Biotechnology; anti-tubulin antibodies were from Serotec (Oxford, UK).

### 3.10. Senescence-Associated β-galactosidase Activity

Staining for SA-β-galactosidase activity was done on cells previously fixed in 3% formaldehyde-cell medium 10% FBS solution. After extensive washing, fixed cells were treated at 37 °C for 24 h with a staining solution as previously described [35]. To this purpose, cells were plated in triplicate at 5 × 10^4^ in 35-mm dishes. Adherent, SA-β-galactosidase-positive cells were searched in random fields using bright field microscopy.

### 3.11. Statistical Analysis

All tests described therein have made use of the free statistical package GraphPad [36]. Significance was assessed using Student’s *t* test for comparison between two means (*p* values: * < 0.1; ** < 0.01; *** < 0.001).

### 3.12. Chemicals

Column chromatography (CC) was carried out on Merck Kieselgel 60 (230−400 mesh). Electronic Impact Mass Spectra (EI-MS) were obtained with a QP-5050A (Shimadzu) EI 70 eV spectrometer. Anal. TLC: Kieselgel 60 F254 plates (0.2-mm; Merck, Darmstadt, Germany); visualization under UV light and by spraying with H_2_SO_4_/AcOH/H_2_O 1:20:4, followed by heating for 5 min at 110 °C. Liquid chromatography (LC) analysis was carried out using UFLC prominence series (Shimadzu Corp., Kyoto, Japan), equipped with a vacuum degasser, a quaternary pump, an autosampler, a column heater and PDA detector (diode array detector, DAD). Separation was accomplished using a Phenomenex Luna C18 column (Phenomenex, Italy) (5.0 μm, 2.0 cm × 30 cm).

### 3.13. Extraction and Isolation Procedures

**Scheme 1:**
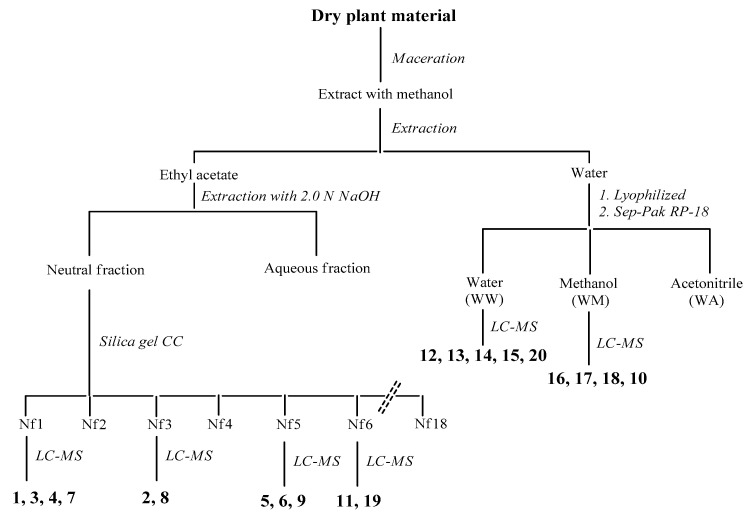
Separation procedures of compounds **1**–**20** of *C*. *coggygria*.

**Figure A3:**
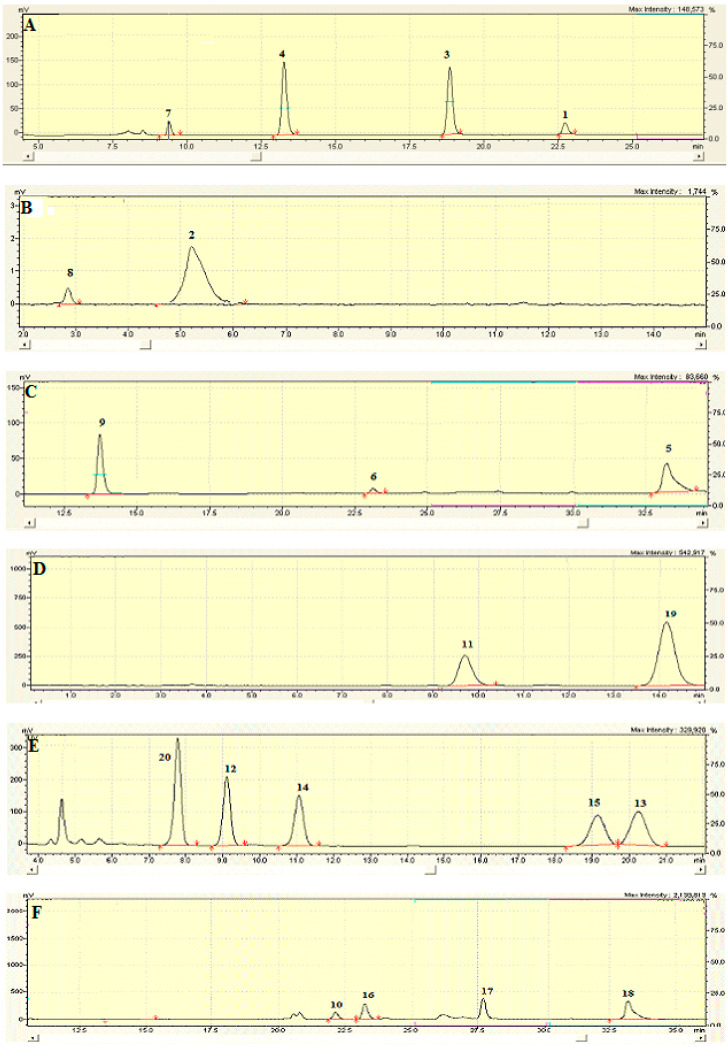
HPLC profiles of compounds isolated from *C. coggygria*: (**A**–**F**) fractions Nf1, Nf3, Nf5, Nf6, WW and WM, respectively.

*C. coggygria*: Dried branches and leaves were air-dried and pulverized. The powder (126 g) was extracted with methanol (3 × 500 mL) for 48 h by maceration. The resultant extracts were combined, filtered and concentrated to dryness *in vacuo* at 40 °C. The resulting extract (6.2 g) was then suspended in water and partitioned with ethyl acetate to give, upon drying, an ethyl acetate-soluble residue (1.9 g first collection; 0.5 g second collection) and water. The organic fraction was fractionated into acidic and neutral fractions with aqueous 2 N NaOH solution. The neutral solution was washed with water and concentrated *in vacuo* (0.52 g), while the aqueous solution was first acidified with aqueous 2 N HCl to neutral pH and then extracted with ethyl acetate, according to Scheme 1 (see also Figure A3).

The neutral fraction was subjected to silica gel column chromatography using a gradient solvent system (CH_2_Cl_2_/acetone = 100:0–95:5–90:10–80:20–70:30–50:50) to give 18 fractions. The fractions cleaner on TLC (Nf1, Nf3, Nf5, and Nf6) were injected to HPLC-MS. Compounds **1**, **3**, **4**, and **7** were identified from the fraction Nf1, compounds **2** and **8** from the fraction Nf3, compounds **5**, **6**, and **9** from the fraction Nf5 and, finally, compounds **11** and **19** from the fraction Nf6. A volume equal to one-tenth of water was lyophilized (320 mg) and filtered on Sep-Pak C18, eluting with water, methanol and acetonitrile. The first two fractions were analyzed by LC-MS and compounds **12**–**15** and **20** were identified from the water fraction and compounds **10**, **16**–**18** were identified from the methanol fraction (Figure A1, Table A1). For comparison with the data reported in the literature and in the database of the detector, all isolated compounds (**1**–**20**) were identified (Table A3).

**Scheme 2:**
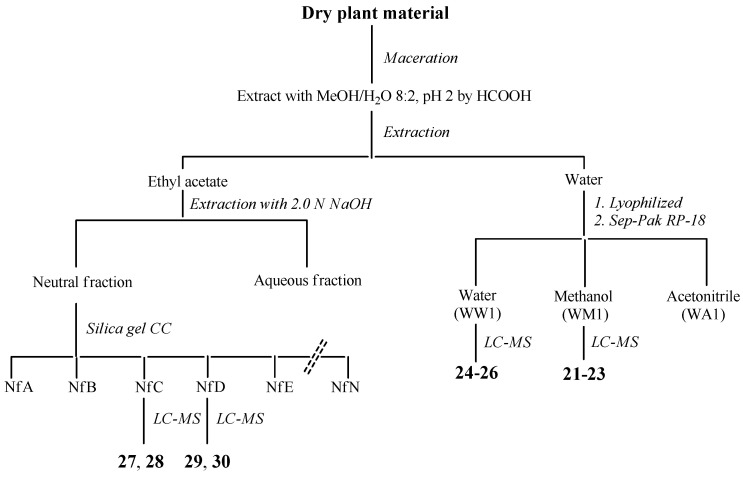
Separation procedures of compounds **21**–**30** of *J*. *communis*.

**Figure A4:**
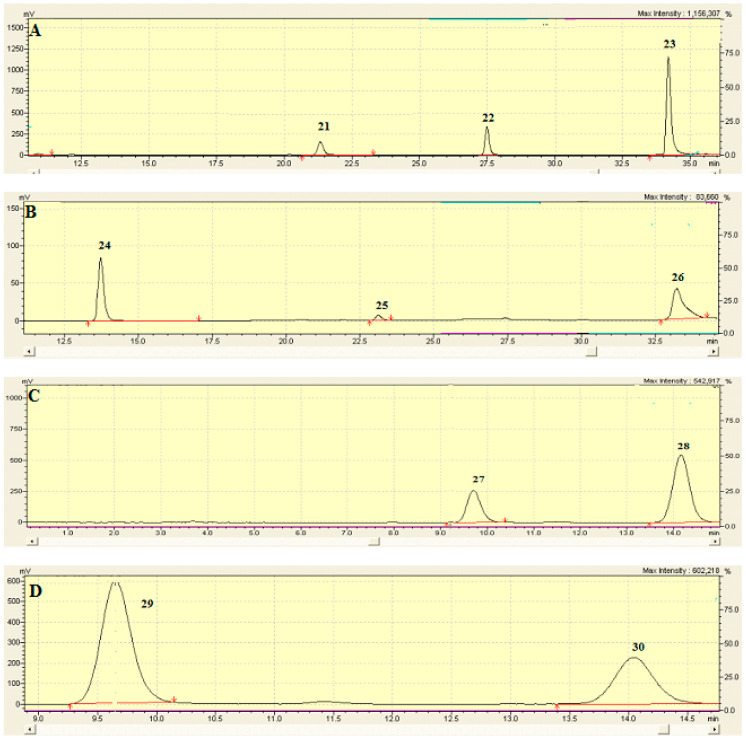
HPLC profiles of compounds isolated from *J. communis*: (**A**–**D**) fractions NfC, NfD, WW1 and WM1, respectively.

*J. communis*: The whole plant (320 g), dry and powdered, was extracted with methanol/water 8:2 at pH 2 by formic acid (3 × 500 mL) for 2 h by maceration. The resultant extracts were combined, filtered and concentrated to dryness *in vacuo* at 40 °C. The resulting extract (22 g) was then suspended in water and partitioned with ethyl acetate to give, upon drying, an ethyl acetate-soluble residue (12 g) and the water (9.5 g). The organic fraction was fractionated into acidic and neutral fractions with aqueous 2 N NaOH solution. The neutral solution was washed with water and concentrated *in vacuo* (7.5 g), while the aqueous solution was first acidified with aqueous 2 N HCl to neutral pH and then extracted with ethyl acetate, according to Scheme 2 (see also Figure A4).

The neutral fraction was subjected to silica gel column chromatography using a gradient solvent system (0.1% formic acid/water/acetonitrile = 90:10–75:25–50:50–25:75–0:100) to give 12 fractions (NfA-NfN). The fractions were directly analyzed by HPLC-MS. Compounds **27** and **28** were identified from the fraction NfC, compounds **29** and **30** from the fraction NfD. A volume equal to one-tenth of water was lyophilized (312 mg) and filtered on Sep-Pak C18, eluting with water, methanol and acetonitrile. The first two fractions were analyzed by LC-MS and compounds **24**–**26** were identified from the water fraction (WW1) and compounds **21**–**23** were identified from the methanol fraction (WM1) (Figure A2, Table A2). For comparison with the data reported in the literature and in the database of the detector, all isolated compounds (**21**–**30**) were identified (Table A4).

### 3.14. Determination of 2,2-Diphenyl-1-picrylhydrazyl (DPPH) Radical Scavenging Capacity

Alcoholic extracts of *J*. *communis* and *C*. *coggygria* were evaluated at different concentration levels. Tests have been carried out performing three replicate measurements on three samples (*n* = 3) of each extract. Results are expressed as the mean ± SD values. In order to estimate the DPPH• scavenging capability, the extracts at various final concentrations (namely 0.625, 1.25, 2.5, 5.0, 12.5, 25.0, 50.0 and 100.0 μg/mL) were dissolved in a DPPH• ethanol solution (9.4 × 10^−5^ M; 1.0 mL) at room temperature. After 30 min, the absorption at 517 nm was measured by a Jasco UV-Vis V560 spectrophotometer in reference to a blank. The results were expressed in terms of the percentage decrease of the initial DPPH radical absorption by the test samples.

### 3.15. LC-MS/MS Analysis

About 1 mg of Nf1, Nf3, Nf5, Nf6, NfC, NfD, WW, WM, WW1 and WM1 fractions were dissolved in 0.8 mL MeOH filtered through a 0.45 mm filter and subjected to high performance liquid chromatography (HPLC). For qualitative analysis, the Shimadzu (Milano, Italy) LCMS-8040 Triple Quadrupole Liquid Chromatograph Mass Spectrometer (LC-MS/MS) was used. Separations were accomplished on Phenomenex Luna C18 column (100 mm × 2.1 mm, 1.6 μm) at a flow rate of 0.25 mL/min with water (solvent A) and acetonitrile (solvent B containing 0.1% formic acid) as mobile phase, and the gradient elution program was as follow: 15%–100% B over 10 min, followed by isocratic elution with 100% solvent (B) from 10–20 min, then returned to 15% from 22 min at a flow rate of 0.8 mL/min. The column temperature was maintained at 45 °C and the injection volume was 10 μL. Separation of compounds was monitored with DAD at 254 and 190 nm and with a mass spectrometry detector. Mass spectrometric analysis (ESI) was carried out on LC-MS 8030 triple-quadrupole mass spectrometer (Shimadzu, Kyoto, Japan). Liquid chromatography–tandem mass spectrometry (LC-MS/MS) was set in the negative and positive ionization mode with spectra acquired over a mass range of 50–1000 *m*/*z*. The acquisition parameters were as follows: interface voltage, 4.5 kV; interface temperature, 230 °C; desolvation line temperature, 230 °C; heat block temperature, 350 °C; desolvation gas, nitrogen; desolvation gas flow rate, 3.5 L/min; drying gas, nitrogen; drying gas flow rate, 20 L/min; collision gas, argon; dwell time, 20 ms; and collision gas pressure, 240 kPa. The most appropriate precursor ion, daughter ion, cone volt-age, collision energy (CE) were adjusted according to each analyte.

## 4. Conclusions

The present findings confirm that the natural blend of compounds in the alcoholic extracts from *J. communis* and *C. coggygria*, have cell cycle specific inhibitory properties that can be conveniently used for *in vitro* studies and encourage efforts for *in vivo* assessments for their potential targeted applications in cancer therapy.

## Figures and Tables

**Table 1:** Major components from *C. coggygria* and *J. communis* extracts.

Ethyl Acetate Extracts		Water Extracts	
*C. coggygria*	*J. communis*	*C. coggygria*	*J. communis*
Liquiritigenin (**1**)	Amentoflavone (**27**)	Rutin (**10**)	Catechin (**21**)
Fustin (**2**)	Cupressoflavon (**28**)	Gallic acid (**12**)	Apigenin (**22**)
Naringenin (**3**)	Corchoionoside C (**29**)	Coumaric acid (**13**)	Quercetin (**23**)
Taxifolin (**4**)	Matairesinol (**30**)	Caffeic acid (**14**)	Isoquercetin (**24**)
Isoliquiritigenin (**5**)		Ferulic acid (**15**)	Apigetrin (**23**)
Butein (**6**)		Rosmarinic acid (**16**)	Kaempferol-7-*O*-α-d-Glucopiranoside (**26**)
Fisetin (**7**)		Chlorogenic acid (**17**)	
Eriodictyol (**8**)		Resveratrol (**18**)	
Myricetin (**9**)		Pentagalloyl glucose (**20**)	
Kaempferol (**11**)			
Sulfuretin (**19**)			

**Table 2:** Bacterial strains in extracts.

Extracts (Undiluted Stocks) 15 µL	Incubation Time (h) (in DMEM)	Bacterial Strain
*J. communis*	72	Sterile
*C. coggygria*	24	*B. licheniformis B. subtilis B. sonorensis*
*P. scolopendrium*	72	*B. licheniformis B. subtilis*
*E. hyemale*	72	*B. licheniformis B. subtilis*

## Supplementary Materials

Supplementary materials can be accessed at: https://www.mdpi.com/1420-3049/21/4/395/s1.

## Appendix A

**Table A1:** Compounds **1**–**20** identified by methanol extract of *C. coggygria*.

No.	Common Name	IUPAC Name	CAS Number	Ref.
1	Liquiritigenin	4’,7-Dihydroxyflavanone	578-86-9	[37]
2	Fustin	3’,4’,7-Trihydroxyflavanol	20725-03-5	[38]
3	(*R*,*S*)-Naringenin	4’,5,7-Trihydroxyflavanone	67604-48-2	[39,40]
4	Taxifolin	3’,4’,5,7-Tetrahydroxyflavanol	480-18-2	[40]
5	Isoliquiritigenin	*trans*-2’,4,4’-Trihydroxychalcone	961-29-5	[41]
6	Butein	*trans*-2’,3,4,4’-Tetrahydroxychalcone	487-52-5	[38]
7	Fisetin	3’,4’,7-Trihydroxyflavonol	528-48-3	[39]
8	Eriodictyol	3’,4’,5,7-Tetrahydroxyflavonol	4049-38-1	[39]
9	Myricetin	3,5,7,3’,4',5’-Hexahydroxyflavone	529-44-2	[37]
10	Rutin	Quercetin 3-*O*-α-l-rhamnopyranosyl-(1→6)-β-d-glucopyranoside	153-18-4	[37]
11	Kaempferol	3,5,7,4’-Tetrahydroxyflavone	520-18-3	[42]
12	Gallic acid	3,4,5-Trihydroxybenzoic acid	149-91-7	[40]
13	Coumaric acid	4-Hydroxycinnamic acid	25429-38-3	[37]
14	Caffeic acid	3,4-Dihydroxycinnamic acid	331-39-5	[37]
15	Ferulic acid	3-Methoxy-4-Hydroxycinnamic acid	1135-24-6	[37]
16	Rosmarinic acid	(2*R*)-3-(3,4-dihydroxyphenyl)-2-[(*E*)-3-(3,4-dihydroxyphenyl)prop-2-enoyl]oxypropanoic acid	20283-92-5	[43]
17	Chlorogenic acid	3-*O*-Caffeoylquinic acid	327-97-9	[37]
18	Resveratrol	3,4’,5-Trihydroxystilbene	501-36-0	[44]
19	Sulfuretin	3’,4’,6-Trihydroxyaurone	50-99-7	[38]
20	Pentagalloyl glucose	1,2,3,4,6-Pentagalloyl-d-glucose	50678-27-8	[37]

**Table A2:** Compounds **21**–**30** identified by methanol extract of *J. communis*.

No.	Common Name	IUPAC Name	CAS Number	Ref.
21	Catechin	3’,4’,5,7-Tetrahydroxy-2,3-trans-flavan-3-ol	154-23-4	[45]
22	Apigenin	5,7,4’-Trihydroxyflavone	520-36-5	[46]
23	Quercetin	3,5,7,3’,4’-Pentahydroxyflavone	117-39-5	[37]
24	Isoquercetin	3-*O*-β-d-Glucopyranosylquercetin	482-35-9	[37]
25	Apigetrin	Apigenin 7-*O*-β-d-Glucopiranoside	578-74-5	[46]
26	-	Kaempferol-7-*O*-β-d-Glucopiranoside	16290-07-6	[47]
27	Amentoflavone	Didemethyl ginkgetin	1617-53-4	[48]
28	Cupressoflavon	-	-	[48]
29	Corchoionoside C	(6*S*,9*S*)-Roseoside A	185414-25-9	[49]
30	Matairesinol	Dibenzylbutyrolactone lignanolide	580-72-3	[50]

**Table A3:** LC-MS product ions Compounds **1**–**20**.

Compound	Precursor Ion (*m*/*z*)	Daughter Ion (*m*/*z*)	Ref.
**1**	255 [M − H]^−^	135	[37]
**2**	271 [M − H]^−^	-	[38], standard
**3**	271 [M − H]^−^	177, 151, 119, 107	[39,40], standard
**4**	303 [M − H]^−^	285, 251, 235, 217, 179, 177, 125, 113, 101	[40]
**5**	279 [M + Na]^+^	-	[41]
**6**	271 [M − H]^−^	-	[38]
**7**	285 [M − H]^−^	257, 241, 229, 163, 135,	[39]
**8**	287 [M − H]^−^	151, 135, 125, 107	[39]
**9**	317 [M − H]^−^	271, 245, 179, 152, 151, 137, 124	[40]
**10**	609 [M − H]^−^	301, 300	[37]
**11**	285 [M − H]^−^	257, 229, 213	[42]
**12**	169 [M − H]^−^	125, 127, 107	[40]
**13**	163 [M − H]^−^	119	[37]
**14**	179 [M − H]^−^	135, 117	[37]
**15**	193 [M − H]^−^	178, 149, 134	[37]
**16**	359 [M − H]^−^	197, 160	[43]
**17**	353 [M − H]^−^	191, 179, 173, 161, 135	[37]
**18**	227 [M − H]^−^	185, 159, 158, 143, 119,	[44]
**19**	269 [M − H]^−^	-	[38]
**20**	939 [M − H]^−^	787, 769, 617	[37]

**Table A4:** LC-MS product ions compounds **21**–**30**.

Compound	Precursor Ion (*m*/*z*)	Daughter Ion (*m*/*z*)	Ref.
**21**	289	248, 227, 217, 203, 188, 164, 151, 125, 123	[45]
**22**	269	227, 195, 183, 151, 121, 117, 107	[46]
**23**	301	273, 229, 178, 151	[37]
**24**	463	300, 301, 303	[37]
**25**	431	269, 225, 197, 183, 169, 151, 121, 117	[46]
**26**	447	285, 254	[47]
**27**	537		[48], standard
**28**	537	-	[48], standard
**29**	385		[49]
**30**	357	342, 313, 298, 209	[50]
