# Correction: Pollio et al. Polyphenolic Profile and Targeted Bioactivity of Methanolic Extracts from Mediterranean Ethnomedicinal Plants on Human Cancer Cell Lines. *Molecules* 2016, *21*, 395

**DOI:** 10.3390/molecules31091498

**Published:** 2026-04-30

**Authors:** Antonino Pollio, Armando Zarrelli, Valeria Romanucci, Alfredo Di Mauro, Federica Barra, Gabriele Pinto, Elvira Crescenzi, Emanuela Roscetto, Giuseppe Palumbo

**Affiliations:** 1 Department of Biology, University of Naples “Federico II”, Complesso di MS Angelo, 80126 Naples, Italy; 2 Department of Chemical Sciences, University of Naples “Federico II”, Complesso di MS Angelo, 80126 Naples, Italy; armando.zarrelli@unina.it; 3 Consorzio Interuniversitario Sannio Tech, P.zza San G. Moscati 8, SS Appia km 256, 82030 Apollosa, BN, Italy; 4 Department of Molecular Medicine and Medical Biotechnology, University of Naples “Federico II”, 80131 Naples, Italy; 5 Institute of Experimental Endocrinology and Oncology (IEOS), National Research Council (CNR), Via S. Pansini 5, 80131 Naples, Italy; e.crescenzi@ieos.cnr.it

**Error in Figure**

In the original publication [1], there was a mistake in the caption of Figure 9 as published. In Figure 9B, right panel, loading control for MCF-7 blot was inadvertently labeled as Tubulin, whereas it should be Actin. The corrected Figure 9 caption appear below.

**Figure 9.** (**A**) Changes in the expression of CDK2 in A549 and MCF-7 cells induced by extracts #57 and #46. The CDK2 doublet visible in the controls fades with the addition of both extracts indicating a decline in the kinase activity. Tubulin has been used as loading control; (**B**) p21 expression in MCF-7 is induced by extracts #46 and #57. Actin has been used as loading control; (**C**) p27 expression (after 24 h) in A549 and MCF-7 cells treated with extracts #46 and #57. p27 protein is significantly induced by both extracts in A549 cells, but not in MCF-7 cells. Actin has been used as loading control. p21 ((**B**), right panel) and p27 ((**C**), right panel) expression in MCF-7 cells were probed from the same blot, and the same actin blot is shown as a control for both p21 and p27.

**Text Correction**

A correction has been made to 3. Materials and Methods, 3.8. Western Blot Analysis:

Filters were washed and stained with specific primary antibodies and then with secondary antisera conjugated with horseradish peroxidase diluted 1:2000 (Bio-Rad). Filters were developed using the enhanced chemiluminescence Western blotting detection reagent (Amersham). Membranes were stripped and re-probed with different antibodies.

The authors state that the scientific conclusions are unaffected. This correction was approved by the Academic Editor. The original publication has also been updated.

## References

[1] Pollio A., Zarrelli A., Romanucci V., Di Mauro A., Barra F., Pinto G., Crescenzi E., Roscetto E., Palumbo G. (2016). Polyphenolic Profile and Targeted Bioactivity of Methanolic Extracts from Mediterranean Ethnomedicinal Plants on Human Cancer Cell Lines. Molecules.

