# Supplementary material for: Polyphenolic Profile and Targeted Bioactivity of Methanolic Extracts from Mediterranean Ethnomedicinal Plants on Human Cancer Cell Lines
Source: Molecules. 2016 Mar 23;21(4):395. doi: 10.3390/molecules21040395 (PMC6274438; doi:10.3390/molecules21040395)
Supplement: Supplementary file 1 [file molecules-21-00395-s001.pdf]

Supplementary Materials: Polyphenolic Profile and Targeted Bioactivity of Methanolic Extracts from Mediterranean Ethnomedicinal Plants on Human Cancer Cell Lines

Antonino Pollio, Armando Zarrelli, Valeria Romanucci, Alfredo Di Mauro, Federica Barra, Gabriele Pinto, Elvira Crescenzi, Emanuela Roscetto and Giuseppe Palumbo.

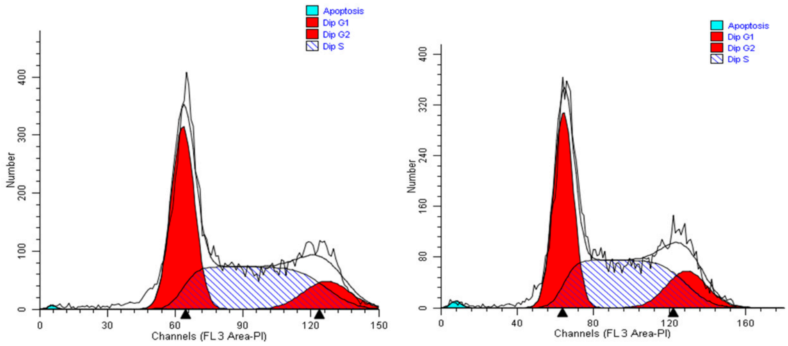

Figure S1. The two profiles of A549 cells treated for 24 hours with extracts #32 (*P. scolopendrium*) and #29 (*E. hyemale*) (0.15%, *v*/*v*) are essentially indistinguishable from the control (see Figure 4, left panel in the text) suggesting a substantial lack of effects on cell cycle.

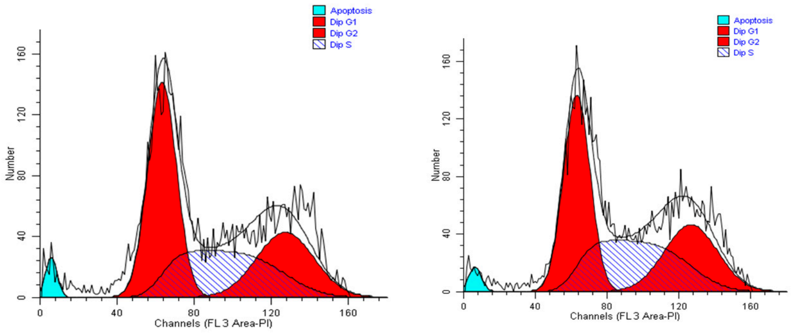

Figure S2. The profiles of MCF-7 cells treated for 24 hours with extracts #32 (*P. scolopendrium*) and #29 (*E. hyemale*) (0.15%, *v*/*v*). In both cases, cells appear to accumulate in the G1 phase, but the differences are not dramatically evident.

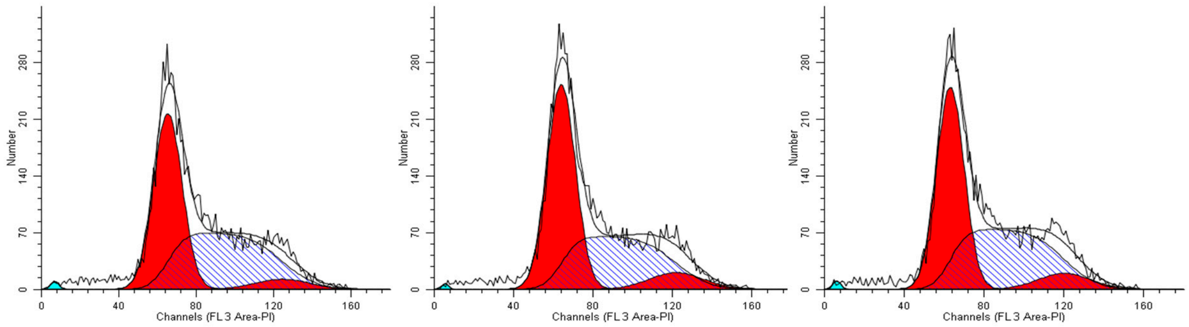

Figure S3. Profiles of untreated U-937 cells (control, left panel) and cells incubated for 24 hours with 0.15%, *v*/*v* of extracts #32 (*P. scolopendri 0.75 cmum*, middle panel) and #29 (*E. hyemale*, right panel). The extracts do not appear to induce visible changes in the respective cell cycle profiles.

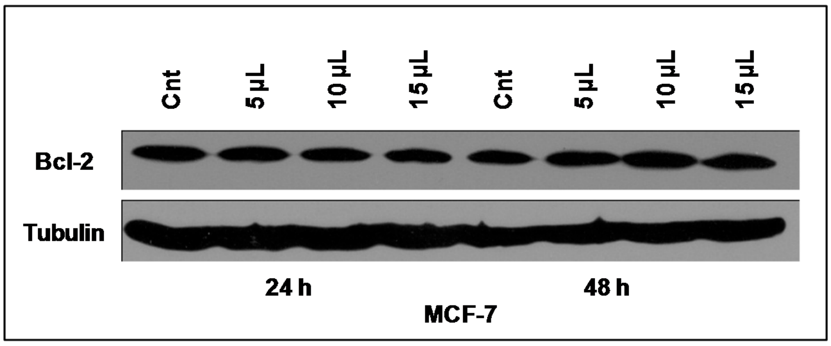

Figure S4. Expression levels of Bcl-2 in MCF-7 cells incubated with extract #46 for 24 and 48 h. No effect was observed even prolonging the incubation time up to 48 hours. Tubulin was used as loading control.

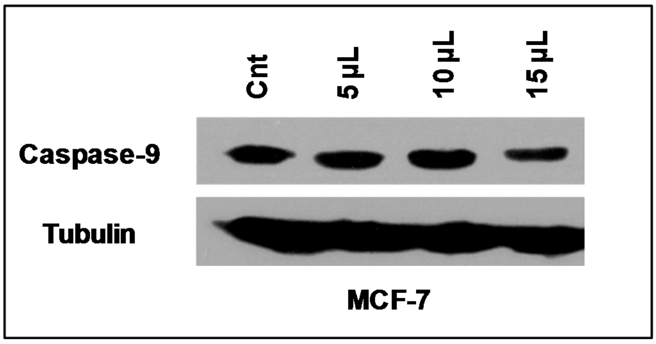

Figure S5. Caspase 9 expression levels in MCF-7 cells incubated for 24 hours with increasing amounts of extract #46. Tubulin was used as load control.
